# Metal and complementary molecular bioimaging in Alzheimer's disease

**DOI:** 10.3389/fnagi.2014.00138

**Published:** 2014-07-15

**Authors:** Nady Braidy, Anne Poljak, Christopher Marjo, Helen Rutlidge, Anne Rich, Tharusha Jayasena, Nibaldo C. Inestrosa, Perminder Sachdev

**Affiliations:** ^1^Faculty of Medicine, Centre for Healthy Brain Ageing, School of Psychiatry, University of New South WalesSydney, NSW, Australia; ^2^Bioanalytical Mass Spectrometry Facility, Mark Wainwright Analytical Centre, University of New South WalesSydney, NSW, Australia; ^3^Faculty of Medicine, School of Medical Sciences, University of New South WalesSydney, NSW, Australia; ^4^Solid State and Elemental Analysis Unit, Mark Wainwright Analytical Centre, University of New South WalesSydney, NSW, Australia; ^5^Faculty of Biological Sciences, Centre for Ageing and Regeneration, P. Catholic University of ChileSantiago, Chile; ^6^Euroa Centre, Neuropsychiatric Institute, Prince of Wales HospitalSydney, NSW, Australia

**Keywords:** LA-ICPMS, metals, Alzheimer's disease, bioimaging, MALDI, FTIR

## Abstract

Alzheimer's disease (AD) is the leading cause of dementia in the elderly, affecting over 27 million people worldwide. AD represents a complex neurological disorder which is best understood as the consequence of a number of interconnected genetic and lifestyle variables, which culminate in multiple changes to brain structure and function. These can be observed on a gross anatomical level in brain atrophy, microscopically in extracellular amyloid plaque and neurofibrillary tangle formation, and at a functional level as alterations of metabolic activity. At a molecular level, metal dyshomeostasis is frequently observed in AD due to anomalous binding of metals such as Iron (Fe), Copper (Cu), and Zinc (Zn), or impaired regulation of redox-active metals which can induce the formation of cytotoxic reactive oxygen species and neuronal damage. Metal chelators have been administered therapeutically in transgenic mice models for AD and in clinical human AD studies, with positive outcomes. As a result, neuroimaging of metals in a variety of intact brain cells and tissues is emerging as an important tool for increasing our understanding of the role of metal dysregulation in AD. Several imaging techniques have been used to study the cerebral metallo-architecture in biological specimens to obtain spatially resolved data on chemical elements present in a sample. Hyperspectral techniques, such as particle-induced X-ray emission (PIXE), energy dispersive X-ray spectroscopy (EDS), X-ray fluorescence microscopy (XFM), synchrotron X-ray fluorescence (SXRF), secondary ion mass spectrometry (SIMS), and laser ablation inductively coupled mass spectrometry (LA-ICPMS) can reveal relative intensities and even semi-quantitative concentrations of a large set of elements with differing spatial resolution and detection sensitivities. Other mass spectrometric and spectroscopy imaging techniques such as laser ablation electrospray ionization mass spectrometry (LA ESI-MS), MALDI imaging mass spectrometry (MALDI-IMS), and Fourier transform infrared spectroscopy (FTIR) can be used to correlate changes in elemental distribution with the underlying pathology in AD brain specimens. Taken together, these techniques provide new techniques to probe the pathobiology of AD and pave the way for identifying new therapeutic targets. The current review aims to discuss the advantages and challenges of using these emerging elemental and molecular imaging techniques, and highlight clinical achievements in AD research using bioimaging techniques.

## Introduction

Alzheimer's disease (AD) is the most common progressive age-related neurodegenerative disorder, affecting about 2% of the population in the developed world (Mattson, 2004). Clinically, AD is characterized by devastating effects such as memory loss and decline in other cognitive abilities resulting in loss of independent functioning (Teri et al., 1989; Baddeley et al., 1991; Terry et al., 1991). Pathologically, AD is characterized by two main pathological hallmarks. These include extracellular amyloid plaques composed of insoluble amyloid beta (Aβ) protein produced by irregular cleavage of the amyloid precursor protein (APP), and intra-neuronal neurofibrillary tangles (NFTs) containing hyperphosphorylated tau protein (Khachaturian, 1985; Joachim et al., 1987; Selkoe et al., 1987; Mirra et al., 1991; Brun and Englund, 2002). Although the exact function of Aβ and APP remains unclear, recent studies suggest that APP may play a crucial role in modulating neuronal survival, neurite outgrowth, synaptic plasticity and cell adhesion (Mattson, 1997). NFTs are not restricted to AD, and are also present in other neurodegenerative diseases such as fronto-temporal dementia (Filley et al., 1994).

AD is a complex multifactorial disorder associated with irregular protein aggregation (Pimplikar et al., 2010). Interestingly, accumulation of Aβ protein has been observed in cognitively normal brain, and sometimes an absence of Aβ deposits has been noted in some postmortem in patients who had been clinically diagnosed with AD (Edison et al., 2007). Moreover, various pathobiological mechanisms that are un-related to amyloid accumulation have been associated with the development and progression of AD. For instance, familial mutations in APP and presenilin-1 have been shown to induce autophagic dysfunction and impaired lysosomal proteolysis, cerebral hypoperfusion, and AD (Lee et al., 2010; Pimplikar et al., 2010; Wong and Cuervo, 2010). Furthermore, excess or deficiency in several nutritional, environmental or genetic factors may also potentiate AD-like pathology, making the etiology of this debilitating disorder difficult to elucidate (Russ et al., 2012).

Metals have a diversity of roles in medical biology encompassing both health and disease states (Olanow and Arendash, 1994; Oteiza et al., 2004; Farina et al., 2013; Jellinger, 2013; Grubman et al., 2014). Metals such as lead and mercury cause well established neuropathologies. By contrast several types of metal ions, such as potassium, sodium and calcium are vital for normal nerve cell function. Several other metals (copper, zinc, iron, magnesium, manganese, cobalt) have functional roles in enzymes and proteins (Yokel, 2006; Molina-Holgado et al., 2007; Farina et al., 2013). For example, brain iron is used by lipid and cholesterol synthesizing enzymes (Bartzokis, 2004) and up to 70% of brain iron is found in association with myelin (de los Monteros et al., 2000; Bartzokis, 2004). However, the careful control of metal ion compartmentalization and usage in the brain is critical, so that metal associated toxicity is avoided. The etiology of several neuropathologies includes a dysfunctional association between otherwise important trace elements and particular proteins or peptides (Table 1). Consequently the pathophysiology of metal-protein interactions in neurodegenerative diseases generally and in AD specifically is an area of growing interest. Divalent metal cations accumulate in plaque deposits and the inflammatory and oxidative processes which are well documented in AD may be mediated through chemistries involving metals (Table 2). However, the biochemistry of metal-protein interactions, sources of accumulating metals and chelation mechanisms are yet to be fully explored in AD.

**Table 1:** **Metal Protein Interactions in Neurodegenerative Diseases**.

**Neurodegenerative disease**	**Metal/s**	**Metal binding protein with a link to neurodegeneration**	**References**
AD	Zn^2+^, Cu^2+^, Fe^2+^, Al^3+^	Zn^2+^, Cu^2+^, Fe^2+^ are sequestered by Aβ fibrils and oligomers leading to oxidative stress.	Rodella et al., 2008; Thinnes, 2010; Savelieff et al., 2013; Watt et al., 2013
		Al^3+^ is potentially involved in the formation of NFTs	
Down's syndrome	Zn^2+^, Cu^2+^, Fe^2+^	Aβ fragment of the amyloid precursor protein associates with a number of divalent metals resulting in amyloid plaque formation	Kedziora et al., 1978; Prasher et al., 1998; Savelieff et al., 2013
Amyotrophic lateral sclerosis (Motor Neuron Disease)	Cu^2+^, Zn^2+^	Mutations in the metalloprotein superoxide dismutase (SOD) are associated with MND	Ince et al., 1994; Divers et al., 2006
Spongiform encephalopathies	Cu^2+^	Prion Protein (Sc)	Basu et al., 2007; Singh et al., 2009, 2012a; Singh and Singh, 2010
Wilson's disease	Cu^2+^	Mutations in ATP7B, a putative Cu^2+^ transporting gene product, leads to decrease in ceruloplasmin and consequent Cu^2+^ accumulation	Peng et al., 2012; Walshe, 2012; Liggi et al., 2013; Ni et al., 2013
Friedreich's ataxia	Fe^2+^	Deficiency of mitochondrial protein frataxin is linked to altered Fe^2+^ homeostasis	Michael et al., 2006; Koeppen et al., 2007; Popescu et al., 2007; Lim et al., 2008
NBIA1 (Hallerverden-Spatz Syndrome)	Fe^2+^	Brain Fe^2+^ deposition possibly in association with the protein synuclein	Valentin et al., 2006
Parkinson's disease	Fe^2+^ Zn^2+^	Aggregates of α-synuclein form and release H_2_O_2_ in the presence of Fe^2+^ Increased localized brain Ferritin levels	Dashdorj et al., 2012; Lucas, 2012; Binolfi and Fernandez, 2013; Björkblom et al., 2013
Aceruloplasminemia	Cu^2+^, Fe^2+^	Mutations in the Cu^2+^ binding metalloprotein ceruloplasmin gene result in accumulation of Fe^2+^ in neurons	Dunaief et al., 2005; Kono et al., 2006; Oide et al., 2006; Gonzalez-Cuyar et al., 2008
Effects of Mn^2+^ in other neurodegenerative diseases	Mn^2+^	Manganism can lead to Huntington's disease and Parkinsonian-like symptoms. The precise mechanism how manganese can damage the CNS is unclear	Bowman et al., 2011

**Table 2:**
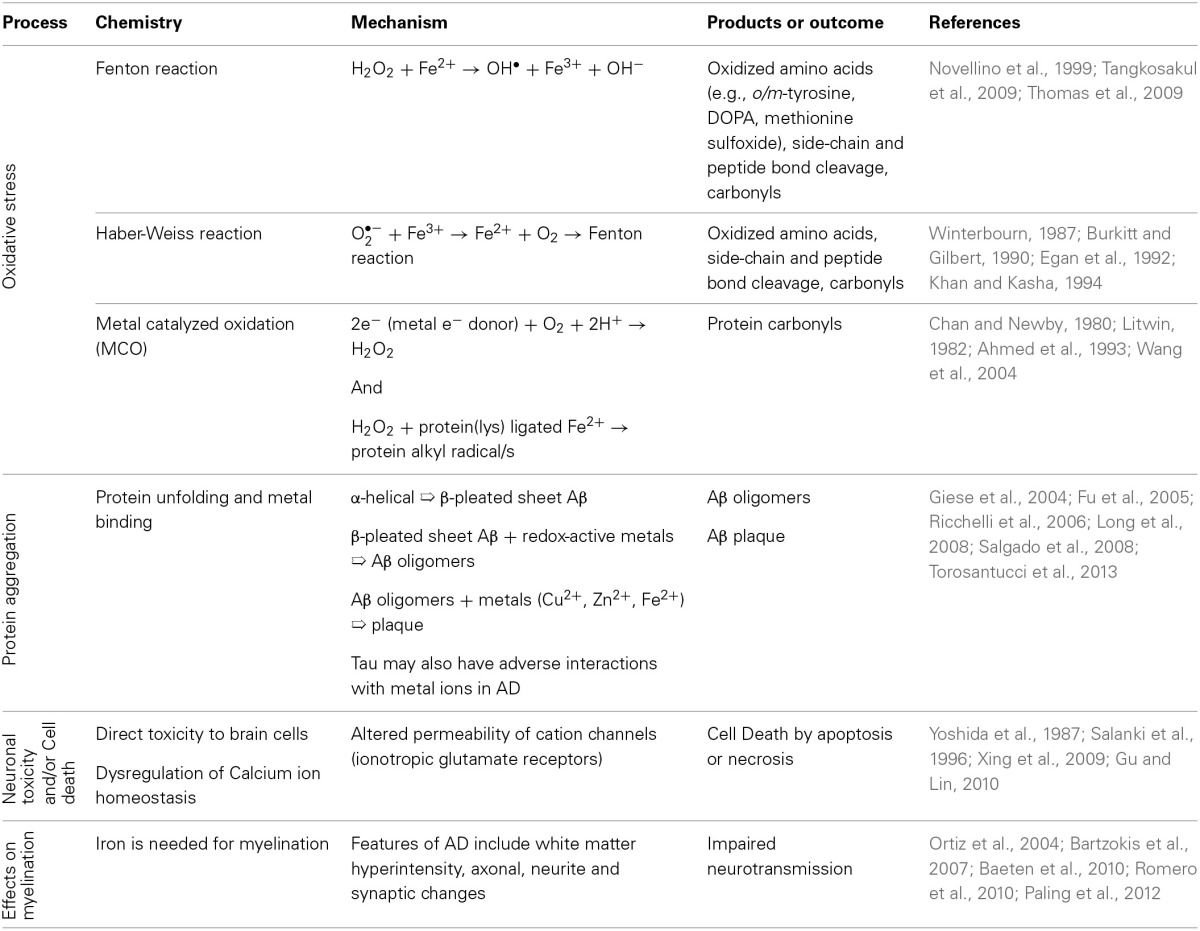
**Processes in the Alzheimer's Disease Brain linked with metals**.

The toxicity of Aβ is linked to changes in its structure from the soluble α-helical form to the insoluble β-pleated sheet form with consequent plaque formation, in which metals such as copper, zinc and iron are sequestered (Lovell et al., 1998a). It is not clear what molecular events trigger plaque formation, a process which may begin much earlier in life than the clinical symptoms of AD (Almkvist and Winblad, 1999). However, dissolution of plaque with metal chelating agents such as clioquinol is a potential new treatment (Cherny et al., 2000, 2001), highlighting the significant role that metals play in the etiology of this disease (Richardson, 2004).

**Figure 1:**
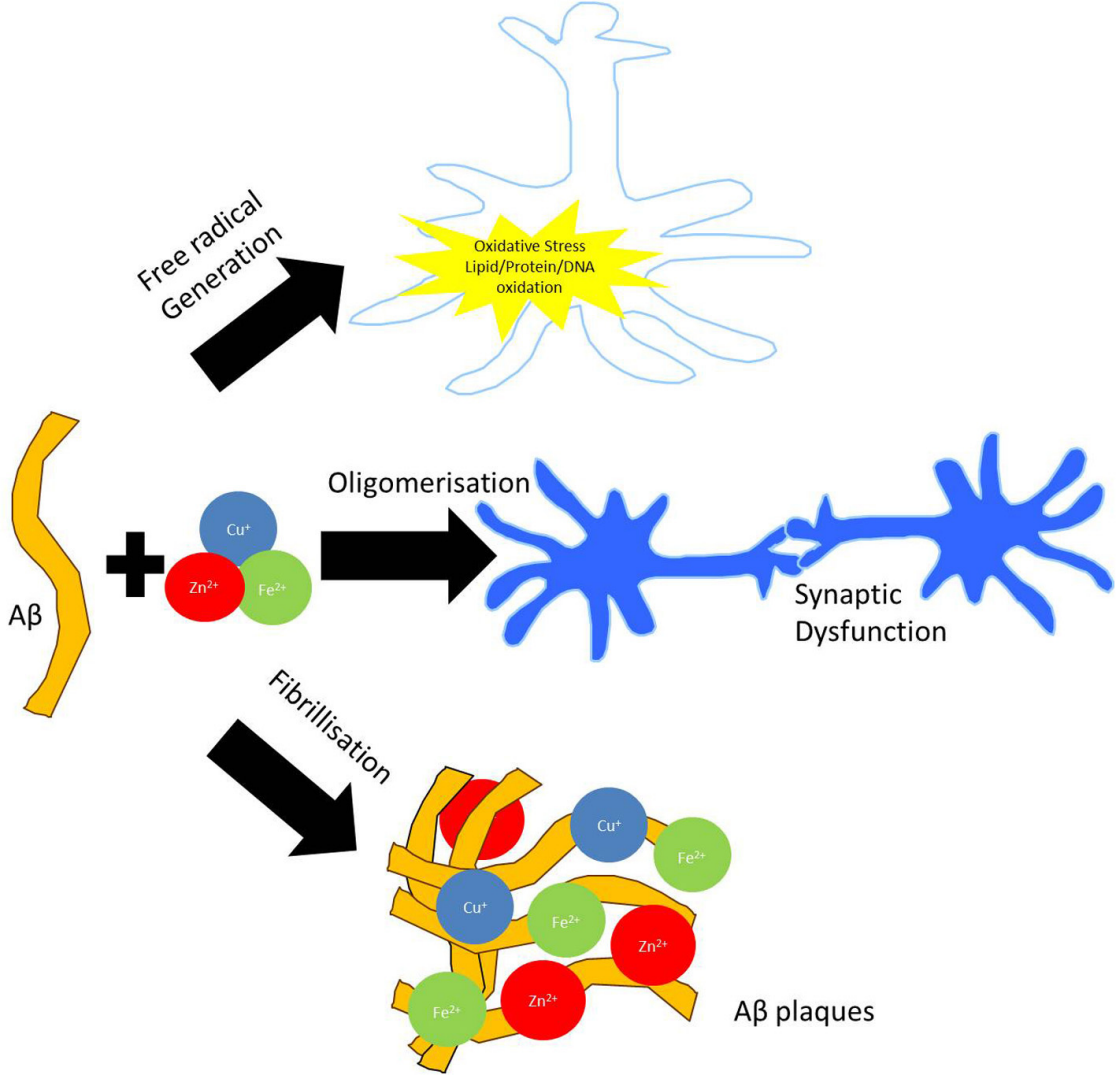
**Involvement of metal dyshomeostasis in AD pathology**. Aggregation of Aβ can bind redox active metals such as copper, iron, and zinc in amyloid plaques. Sequestration of these biometals on Aβ fibrils and oligomers can potentiate synaptic dysfunction. Redox cycling of Cu^2+^/Cu^+^ and Fe^3+^/Fe^2+^ in the amyloid plaques are capable of producing hydrogen peroxide (H_2_O_2_), which can enter the cell. Through Fenton chemistry this can lead to the production of hydroxyl radical (OH^•^) capable of inducing oxidative modifications to both extracellular (i.e., proteins and lipids) as well as intracellular (DNA) macromolecules.

Metal ions, such as those sequestered in plaques, also participate in oxidation and free radical production (Figure 1) (Multhaup et al., 1996). These processes are well documented in AD as are inflammatory processes, mediated by the presence of activated microglia and astrocytes, which generate high levels of Aβ (Busciglio et al., 1993). Metals such as copper, zinc, iron and aluminum have been implicated as possible contributors to neurodegenerative processes. In a few cases, well established links between metals and the function of specific proteins have been demonstrated (Table 1). However, as a subset of all the proteins studied in neuropathology, the metalloproteins are under-represented (Dobson, 2001). Since metal containing active sites of proteins are often involved in oxidation reactions and/or free radical generation, alterations to their biochemistry may be of particular interest in neurodegenerative conditions. Links between protein dysfunction and the role of metals in AD are emerging; (i) divalent metal cations are sequestered in Aβ plaques, (ii) oxidative processes are well documented in AD and metal cations, particularly iron, are a potential source of reactive species. Though metals are likely to play a significant role in AD and other inflammatory diseases, relatively little is known about their sources, mechanisms of transport and chelation, biochemistry and interactions with proteins.

Apart from redox active metals associated with the pathological hallmarks of AD, the presence of other trace metals may also be related to impaired cognitive function in AD. Several toxic heavy metals, including arsenic, lead, mercury, and cadmium are present in the environment due to their high industrial demand (Park et al., 2014). These metals serve no biological function, and their accumulation in the brain is attributed to contact between humans and the environment (Chowdhury and Chandra, 1987). Exposure to arsenic induces neuropathological and behavioral abnormalities similar to clinical features reported in AD and other related neurodegenerative disorders (Gong and O'Bryant, 2010). Lead, which is a well-established neurotoxic pollutant, can induce tau hyperphosphorylation, white matter degeneration, cellular apoptosis, and changes in cellular morphology, and impaired neuronal function (Yun and Hoyer, 2000; Rahman et al., 2012). While cadmium can induce hepatic and renal toxicity, cadmium and lead can also disrupt cholinergic transmission by reducing the turnover of the essential neurotransmitter, acetylcholine (Webster and Valois, 1981; Costa and Fox, 1983; Patra et al., 1999; Singh et al., 2012b). Inorganic mercury can mimic all the pathological hallmarks of AD in animal models (Saxe et al., 1999; Rusina et al., 2006; Mutter et al., 2010). Under normal physiological conditions, sequestration of arsenic, lead, cadmium, and mercury by the lateral choroid plexus represents a protective mechanism to prevent the influx of heavy metals from the blood and into the brain. However, elevated levels of cadmium and mercury can directly damage the choroid plexus, thus limiting the function of this endogenous defense mechanism (Gerhardsson et al., 2011). The toxicity of these metals in human neurodegenerative disorders is dependent on the concentration of the environmental contaminant, and chronic exposure to heavy metals can induce toxicity at relatively low levels (Llobett et al., 2003).

The presence of sequestered biometals such as copper, zinc, and iron in β-amyloid plaques of AD-affected brain tissue, and the presence of toxicological metals as potential pathological cofactors in AD, has led to a focus on metal imaging (Hutchinson et al., 2005; Lelie et al., 2011; Pithadia and Lim, 2012; Stavitski et al., 2013). We should note that not only metals, but a wide range of elements may be imaged, down to ultratrace levels, and at length scales from micron to tens of nanometers. In certain cases isotopes, and even oxidation state and the coordination environment around specific elements can be imaged, potentially increasing the scope of trace element research in neurological disease beyond what has been studied to date. Visualizing changes in element concentration and matching them to anatomical and pathological features enhances our traditional approach to exploring the role of metal ions in neurological disease. Reviews on metal imaging in neurobiology have been presented recently (Bourassa and Miller, 2012) and a comprehensive range of instrumental techniques is available from McRae et al. (2009). However, the field continues to expand rapidly as spatially resolved elemental analysis is now a well-recognized method to investigate chemical changes associated with pathology in biological tissues. The experimental techniques used to obtain elemental information from tissues are quite diverse, with a range of different capabilities in spatial resolution, sensitivity and quantification. This review provides an overview of common instrumental techniques and examples of biological imaging with an emphasis on Alzheimer's studies. Elemental imaging is the main topic of this review; although a selection of molecular imaging examples are presented to demonstrate how these techniques can supplement the elemental bioimaging. Selective colorimetric and fluorescent staining is not covered here, but has been recently reviewed with a focus on neurobiology (Que et al., 2008).

## General overview of elemental imaging techniques for biological tissues

A significant challenge in this field is measuring a specific area on a sample that is small enough to remain biologically relevant, but large enough to enable the elements to be detected. When visual imaging is required to match anatomical features with the elemental distribution, the measurement needs to be carefully planned to leave any destructive analysis as the last step. Most of these techniques will damage the sample to an extent, for example radiation damage in the case of synchrotron techniques, or ablation of the sample surface into gas or ions in the case of laser and ion beam sampling.

It can be instructive to present the most common methods for elemental imaging by their sampling modes, as this will influence the achievable spatial resolution and detection limit. The most common techniques can be classified as (a) ablation of material off the surface that is then directed into an elemental analyzer, (b) ion generation within the sample, and (c) ion generation and ablation from the sample surface. Most of these techniques create spatially resolved elemental data by moving a flat specimen on a stage in precise intervals under the incident beam, and recording the change in analyte flux (ion, electron, photon) that is associated with a specific element.

### Ablation techniques

A pulsed laser can be used to ablate material from a selected area of the sample surface and the gaseous plume swept into another instrument for elemental analysis. A laser ablation (LA) sampling accessory can be integrated with a more traditional atomic spectroscopy system for sensitive multi-elemental analysis. These are usually inductively coupled plasma (ICP) systems using mass spectrometry (LA-ICPMS), or optical emission spectroscopy (LA-ICPOES) for detection of the elements (Qin et al., 2011). Mass spectrometry has the advantage of higher detection limits than optical emission spectroscopy, however there are drawbacks with mass interferences, and the time taken to sweep through the selected ion set, resulting in fewer available ions in order to create an image within a practical length of time. For example, imaging 6 metals across a 4 × 4 mm tissue section with a step size of 30 micron might take 12–24 h (Ketola and Mauriala, 2012). Nevertheless, LA-ICPMS is by far the more common technique for elemental imaging of biometals and toxicological metals in tissues than LA-ICPOES. Another variation on laser sampling is to detect the atomic excitation spectrum directly from the ablated plume, a technique known as laser induced breakdown spectroscopy (LIBS) (Pareja et al., 2013). LA techniques provide excellent analytical sensitivity in atmospheric or relatively low vacuum conditions. However, it is a destructive technique, and delivering sufficient energy to the sample to allow detection tends to limit the spatial resolution. As a result, LA techniques are well suited for analysis of whole tissue sections, but individual cells or pathological features such as amyloid plaques ~20 micron are represented in an image as a single measured point (Hare et al., 2010, 2011; Lear et al., 2012; Chou et al., 2014). Metal imaging of an individual cell or plaque requires the higher resolution available from some of the techniques described below.

### Sample ionization techniques

Highly focused X-rays, electrons, or proton ion beams in a high-vacuum chamber can be used to eject an electron from the core shell of an atom in the sample (Fahrni, 2007). The energy of the ejected electron can be measured using X-ray photoelectron spectroscopy (XPS) to determine the element from which it originated. In certain cases, XPS is able to provide information on oxidation states and the chemical environment around an element, although spatial resolution is limited to 5–50 micron, and detection limits are relatively poor (around 0.1 atomic%), virtually ruling out the technique for trace metal studies (Paunesku et al., 2006). The majority of sample ionization techniques utilize the secondary process where outer shell electrons fill the core shell hole in the ion fluorescing X-rays with a characteristic wavelength for each element in the sample. When atoms are ionized using an electron beam, usually in an electron microscope, the technique is known as energy dispersive X-ray spectroscopy (EDX or EDS), sometimes referred to as electron photon micro analysis (EPMA). If ionization is achieved using an X-ray beam, the technique is X-ray fluorescence microscopy (XFM) or synchrotron radiation micro-X-ray fluorescence (SR-μXRF) (Paunesku et al., 2006; Ralle and Lutsenko, 2009). Ionization can also be performed using a focused beam of protons in a technique called particle induced X-ray emission (PIXE). All of these techniques are performed in high-vacuum environments, so steps such as cryopreservation or careful drying must be taken to protect biological samples or specimen degassing that can reduce the performance of the instrument (de Silva et al., 2006; George et al., 2011; Ramsay et al., 2011; Weekley et al., 2013).

### Secondary ionization techniques

Ablation-ionization directs a highly focused beam of ions, such as oxygen or cesium in the case of secondary ionization mass spectrometry (SIMS), onto a tissue surface under vacuum (Altelaar and Piersma, 2010). This is a destructive process that results in ions being ejected from the surface. The ions are usually detected with a magnetic sector (NanoSIMS) or time-of-flight (TOF SIMS) mass spectrometer (Pacholski and Winograd, 1999; Eller et al., 2013; Fernandez-Lima et al., 2013). A recent review is available detailing the general capabilities of mass spectrometry-ablation techniques such as SIMS (Amstalden van Hove et al., 2010). The ability to focus ion beams down to very small spot sizes enables excellent spatial resolution, with features of 50 nanometers having been reported in the case of the NanoSIMS. However, micron to submicron imaging is more common since, in order to generate sufficient secondary ions for detection with a very small spot size, the ablation depth needs to increase. Submicron imaging at hundreds of nanometers is more common, and is sufficient for cellular differentiation or observing small pathological features (Quintana et al., 2007; Musat et al., 2012). It is notable that the mass spectrometry techniques also enable more specialized imaging of isotopes across a surface, as well as providing more general elemental imaging.

### Other techniques

Electron energy loss spectroscopy (EELS) measures the energy loss due to scattering processes when a low energy, monoenergetic electron beam interacts with a sample. When used in a transmission electron microscope, EELS can provide atomic-scale resolution with excellent detection limits although biological applications are limited (Quintana et al., 2000; Terada et al., 2002). There are a variety of X-ray techniques that have evolved as a result of the high-intensity X-ray sources available at numerous synchrotron facilities around the world. X-ray Absorption Near Edge Structure (XANES), also known as Near edge X-ray absorption fine structure (NEXAFS), is a technique where the element composition change the absorption spectrum of the X-ray beam, providing information on elemental oxidation state and coordination geometry around metal ions (Bourassa and Miller, 2012). Although potentially powerful, imaging of biological materials using this technique is still in development.

Magnetic resonance imaging (MRI) remains the most widely used metal imaging technique in the clinical setting (Helpern et al., 2004). Although recent advances in MRI have made it possible to detect the levels of iron at physiological concentrations, copper and manganese are still not widely detectable, since they are present in low concentrations in the brain (Schenck and Zimmerman, 2004). Current MRI techniques exhibit lower spatial resolution compared to elemental imaging techniques mentioned above, but demonstrate the advantage of imaging live patients rather than cryo-cut postmortem tissue sections (Schenck and Zimmerman, 2004).

Positron emission tomography (PET) is another technique which facilitates *in vivo* medical imaging, usually of small molecules including glucose and more recently Aβ plaques using Pittsburg Compound B (PiB PET). More recently a novel metal imaging PET approach has been developed, using radioactive coordination bis(thiosemicarbazonato)copper complex of ^64^Cu. This targets copper homeostasis and has been designed to bind selectively to amyloid plaques (Hickey et al., 2013). Copper radiolabels are essential for increasing our understanding on of the mechanisms of copper dyshomeostasis in AD.

## Combined bioimaging techniques in tissue sections

Complementary information regarding the role, uptake, transport, and storage of redox active metals associated with irregular protein abnormalities can be obtained using a combination of elemental imaging techniques, such as LA-ICPMS, and other biomolecular mass spectrometry imaging techniques such as laser ablation coupled with electrospray ionization mass spectrometry (LA-ESI-MS) or MALDI-IMS. While LA-ICPMS can be employed to identify the specific protein-bound metals, ESI-MS/MALDI enables the identification of the structure, dynamics and biological function of metal-protein complexes (Becker et al., 2008; Dobrowolska et al., 2008; Jakubowski et al., 2008; Wu et al., 2009).

ESI-MS is an ionization technique that is employed to detect polar compounds within a biological specimen (Fenn et al., 1989). This method is used to identify molecules that do not contain an intrinsic ionizable site through formation of adduct ions. Molecules which exhibit sufficient dipole potential to interact with a small anion or cation can be readily ionized and detected using ESI-MS. It is useful for the detection of triacylglycerols (TAGs) which contain long chain fatty acids. These molecules can be ionized and quantified with sensitivity in the low picomole range due to the formation of lithiated adducts which are formed when chelated lithium ions non-covalently bond with the carbonyl structures that are present in the infused solution (Han et al., 2000; Han and Gross, 2001). The benefits of using ESI-MS include more accurate quantification of lipid classes and subclasses, a greater signal-to-noise ratio in comparison to other mass spectrometry techniques, and an almost linear relationship between the relative intensities of molecular ions and the mass of individual lipids (Han and Gross, 1994).

MALDI-IMS allows the analysis of a diversity of biopolymers with a variety of mass ranges. This approach has a lower spatial resolution but much higher mass range than TOF-SIMS, which is limited to identification of analytes with a molecular mass of less than 1 kDa (McArthur et al., 2004). A variety of analytes can be examined using MALDI-IMS, including metabolites, lipids, proteins, peptides, carbohydrates, and drugs. However, this method is limited by signal suppression effects. For instance, some analytes are more efficiently ionized during MALDI-IMS. These artifacts are not only due to their unique chemical structure, but also to relative amounts present in the biological tissue (Knochenmuss et al., 1998). Alternatively, proteins can be extracted from the tissue section using hydrophobic materials, while preserving their specific location (Chaurand et al., 2004). Adaptation of MALDI-TOF to 2D and even 3D tissue imaging applications has necessitated use of rapid fire long lived lasers, such as the 2 kHz Nd-YAG, to accommodate the need to acquire 1000s of spectra across a tissue section. High end MALDI imaging mass spectrometers currently combine high mass resolution of 40,000 (1 ppm mass accuracy), wider mass range (50–300,000 Da), spatial resolution down to 10 μm, and TOFTOF capabilities for peptide sequencing. This combination of features allows detailed characterization of a diversity of tissue constituents, top-down sequencing of proteins as well as the more commonly used bottom-up techniques involving enzymatic/tryptic digestion and peptide sequencing, analysis of posttranslational modifications such as glycosylation. A growing body of literature recognizes the power of this approach (Cornett et al., 2007; Schuerenberg et al., 2007). A combination of mass spectrometry imaging techniques using LA-ICPMS and detailed proteomics analysis can be performed using thin cryo-cut sections of brain. MALDI-IMS is a relatively non-destructive technique so the tissue remaining after initial proteomic, metabolomic or lipidomic analysis can then be analyzed for elemental composition using LA-ICPMS.

Fourier transform infrared spectroscopy (FTIR) is another molecular imaging tool that can be combined with LA-ICPMS. These tools have been used to image the secondary structure of metal-protein complexes (Haris and Severcan, 1999). FTIR is a non-destructive technique, allowing further analyses to provide complementary information and to show spatial relationships between diverse analytes and/or functional groups, which may provide insight into biological/functional relatedness. The protein's FTIR consists of two main features: the Amide I band (~1650 cm^−1^) which arises from the C=O stretching vibration, and the Amide II band (~1540 cm^−1^) which is due to the N-H bending and C-N stretching vibrations of the peptide backbone. The vibrational frequency of an aggregated protein is about 1620–1625 cm^−1^, owing to its hydrophobic environment (Goormaghtigh et al., 2006; Miller et al., 2006). Apart from examining the protein structure *in vitro*, FTIR can also be used to directly investigate irregular protein misfolding and aggregation both *in vitro* and *in vivo*. Protein aggregates are generally small, ranging from nanometers, to 20–30 μm for larger aggregates. As well, the spectral differences related to changes to protein conformation are subtle, requiring spectra with high signal to noise ratio (Choo et al., 1996; Miller et al., 2006). These difficulties have been resolved using the greater brightness of a synchrotron infrared source to directly assess protein aggregation and misfolding in AD tissue.

## Recent applications of bioimaging in Alzheimer's research

Metals have been shown to be associated with the pathogenesis of AD for over 50 years since the discovery of significant iron deposition in postmortem AD brain tissue using Prussian blue stain (Goodman, 1953). Since then, other redox active metals have been implicated in AD, including copper, zinc, and aluminum. Several metal bioimaging strategies have been utilized to examine the distribution of metals in human clinical AD brain tissue and AD mouse models to better understand the relationship between metal dyshomeostasis and the etiology and progression of AD.

### Metals and Aβ plaques

It has been well established that Aβ plaques are rich in metal ions (Opazo et al., 2002). These relatively high concentrations of metals within the plaques compared to adjacent tissue have been reaffirmed using a variety of bioimaging techniques. PIXE and XFM has been used to show that both the outer and central regions of the Aβ plaques contain elevated levels of iron, copper and zinc in human AD brain specimens (Lovell et al., 1998a,b). Although copper and zinc binding sites are present on the Aβ peptide, iron does not appear to directly interact with Aβ (Atwood et al., 2000; Bush, 2003; Roberts et al., 2012). Recently, synchrotron X-ray absorption, diffraction, and tomography techniques have been used to identify the presence of biogenic magnetite and/or maghematite in the plaque cores, implicating the likely role of a novel biomineralization process to account for the accumulation of iron in Aβ plaques (Collingwood et al., 2005, 2008).

Transgenic mouse models have provided additional advantages over postmortem human clinical AD specimens in the control of both genetics and onset of AD-like symptoms. Using XFM, no abnormal increase in copper or iron were reported in with disease progression in the PSAPP double transgenic mouse which expresses a chimeric mouse/human amyloid precursor protein (Mo/HuAPP695swe) and a mutant human presenilin 1 (PS1-dE9) both directed to CNS neurons. This mouse model develops amyloid pathology as well as learning and memory deficits by 6 months of age, independent of signs of neurodegeneration (Leskovjan et al., 2011). Moreover, only a slight upregulation in zinc concentrations was reported at the late stages of the disease. By contrast, the CRND mouse which expresses two familial mutations in the human Swedish (K595N/M596L) and Indiana (V717F) APP gene exhibited a 2–3-fold increase in the concentration of iron, copper, and zinc in the plaques after 6 months of age using PIXE. This unique mouse model develops diffuse and compact plaques by 10 weeks of age and Aβ deposition continues with advanced age (Rajendran et al., 2009). Similar findings have been reported using LA-ICPMS analysis of plaques present in the brains of TASTPM mice, which carry both the APP K670N/M671L and PS1M146V mutation and develop plaques by 4 months of age (Hutchinson et al., 2005).

### Metal dyshomeostasis in aging and AD

Since ageing is a major risk factor for the development of AD, examining the age-related changes in metal distribution is critical for understanding the role that metals play during pathological and physiological conditions. Using LA-ICPMS, one study showed that iron levels were increased in the “physiologically” aged brain of a non-transgenic mice (14 months) compared to a young (2 month) mice (Becker et al., 2010). These increases were observed in the substantia nigra, thalamus, and the CA1 region of the hippocampus which are associated with development of neuropathologies. Remarkably, zinc levels remained unchanged and zinc-enrichment in the CA3 of the hippocampus was already detected in young mice. This may be associated with the important role of zinc as an essential neuro-co-transmitter that is released from synaptic vesicles (Becker et al., 2010).

Evidence of metal dyshomeostasis has also been reported in AD. Studies using PIXE have shown increased levels of zinc in the amygdala, hippocampus and neuropils of human AD brains (Danscher et al., 1997; Lovell et al., 1998a,b). This is likely to be associated with the increased distribution of zinc enriched neurons (ZEN) which are located in these regions. ZENs maintain intracellular pools of zinc which is necessary as a neuromodulator and neuro-co-transmitter. One hypothesis suggests that zinc released from these neurons can interact with Aβ and promote aggregation (Bush et al., 1994; Frederickson et al., 2005). Zinc deficiency can also lead to excitotoxicity and neurodegeneration (Sensi et al., 2009). Moreover, zinc reuptake is an energy dependent process, and mitochondrial dysfunction can lead to increased free zinc which can interact with Aβ and lead to further neurotoxicity (Mony et al., 2009).

Altered iron levels have also been suggested to play a prominent role in ageing and AD. Iron levels have been shown to increase in the substantia nigra, motor rotex, hippocampus, basal ganglia, putamen, cerebellum and cortex of human normal subjects during ageing (Connor et al., 1992; Deibel et al., 1996; Bartzokis et al., 2000). A similar increase was also reported iron, copper and zinc content was also reported in the PSAPP mouse model in the cortex and hippocampus, and coincided with increased plaque formation using XFM (Leskovjan et al., 2011). Ferritin, the main protein responsible for iron storage, has been shown to increase in the coronal region of human AD plaques using TEM and NanoSIMS (Quintana et al., 2006). It is likely that ferritin, which stores inactive iron (III) under normal physiological conditions may bind redox active iron (II) in the AD brain leading to cell death via oxidative stress.

### Metals and NFTs

Metal dyshomeostasis may also play a role in the formation of NFTs. A 10-fold increase in iron and a 6-fold increase in copper, with a smaller increase in zinc, have been previously reported in NFTs (Morawski et al., 2005). Furthermore, hyperphosphorylated tau, which forms paired helical filaments (PHFs) that lead to NFTs, contains several binding domains which demonstrate some affinity to copper, and the presence of copper can enhance the formation of NFTs (Ma et al., 2006). Iron (III) can also induce NFT formation similar to copper (Yamamoto et al., 2003). Apart from copper, iron and zinc, aluminum has also been associated with the development of AD since it was first identified in neurons with NFTs (Perl and Brody, 1980). However, increased aluminum is also present in non-diseased brain tissue fixed with osmium tetroxide, which contains aluminum (Tokutake et al., 1995; Makjanic et al., 1997). Further work is warranted to validate the involvement of aluminum in AD.

### Lipidomic studies using ESI/MS

ESI-MS techniques have been used to investigate the lipidome in patients with dementia. These studies have demonstrated specific changes to the lipidome in the postmortem gray and white matter in the frontal, temporal and parietal cortex at the earliest clinically-recognizable stage of AD compared to cognitively normal control (Han et al., 2001, 2002). Specifically, plasmenylethanolamine (PlsEtn) mass was reduced by up to 40 mol% of total plasmalogens, in white matter in early AD subjects compared to age-matched controls. PlsEtn mass levels were depleted by 10% in the gray matter in patients with severe AD. Sulfatides, which form specialized components in the myelin sheath which encapsulate neurons, were depleted by 93 and 58 mol% in gray and white matter, respectively, in AD patients in all brain regions that were investigated (Han et al., 2001, 2002). Additionally, a significant increase (>3 fold) in ceramide content was observed in the white matter of all investigated brain regions during early AD. No significant changes have been observed in the levels of other lipid classes, including phosphatidylglycerols, phosphatidylinositols, phosphatidylserines, and phosphatidic acids in early stages of AD although significant reduction (~ 15 mol%) of these lipids occurred in severe AD cases (Han et al., 2001, 2002). Taken together, these results suggest that changes to the lipidome may play a vital role in the pathogenesis of AD and may be associated with early molecular and cellular events which occur in the development of AD, such as neurodegeneration and synaptic dysfunction.

### Maldi-MS imaging in AD

Recently, MALDI-MS has been used to examine the spatial distribution and molecular contents of Aβ plaques. One study showed that Aβ-(1–40) and Aβ-(1–42) are the most abundant amyloid peptides in APP23 transgenic mice encoding the hAPP751 with Swedish mutation (Rohner et al., 2005). In support of this work, other studies have shown that vascular amyloid is primarily composed of Aβ-(1–40) and Aβ-(1–42) (Miller et al., 1993). Additionally, Aβ-(1–40) is the major peptide that is found in aqueous cerebral cortical extracts from AD brains. By contrast, the insoluble amyloid Aβ-(1–42) peptide is primarily localized in the senile plaque cores. Therefore, MALDI-MS can not only be used to identify known targets, but also facilitates mapping of the different peptide targets with high precision and accuracy, that is otherwise not possible when examining whole-brain extracts (Rohner et al., 2005).

### FTIR spectroscopic imaging in AD

In AD, Aβ plaques are formed by the transformation of Aβ from a soluble form through to an oligomeric intermediate, culminating in the formation of an aggregated, fibrillary structure (Ii, 1995). The molecular mechanism which mediates the structural changes and cytotoxicity of Aβ during the aggregative process has been previously investigated in several *in vitro* studies using dichroism (CD) and nuclear magnetic resonance (NMR) to show the structural conversion of Aβ from a soluble α-helical protein to a fibrillar β-sheet protein (Barrow et al., 1992; Zhang and Rich, 1997). FTIR spectroscopy has been essential to examine the specific alignment of β-sheet strands within Aβ fibrils. A study by Petty and Decatur (2005) showed that β-sheets are antiparallel and in alignment across all strands (Petty and Decatur, 2005). Recently, it has been suggested that oligomeric Aβ is more neurotoxic than Aβ fibrils and can form pore-like structures in lipid membranes that can disrupt ion homeostasis, culminating in cell death. FTIR spectroscopy has shown that Aβ oligomers exhibit an antiparallel β-sheet structure, which is closely related to that of bacterial outer membrane porins (Komatsu et al., 2007).

Apart from the Aβ protein, FTIR spectroscopy has also been used to gain a greater understanding of the structural conformation of the tau protein, which is hyperphosphorylated in AD, leading to the formation of NFTs. *In vitro* FTIR analysis provided confirmatory evidence that soluble tau protein is natively unfolded and composed of random coil structures, whilst PHFs which are present in the AD brain have a greater level of β-structure (Berriman et al., 2003). These results provide evidence to support the hypothesis that the repeat domain of tau (which is located within the core of PHFs) displays an enhanced level of β-structure during aggregation, while the N- and C-terminal domains which venture away from the central PHF core are largely random coils (Barghorn et al., 2004).

## Conclusion

Metal imaging techniques are currently primed to facilitate an understanding of the pathobiology of AD, as well as identifying novel diagnostics and therapeutics. Bioimaging techniques are important for elucidating the role of metals in neurodegenerative diseases generally and AD in particular. Advancements in methodology and improved spatial resolution and detection sensitivities are essential for greater insight into the localization and distribution of metal ions at the cellular and tissue level, and their role in disease development and progression. A combination of other imaging techniques such as ESI- and MALDI-IMS, FTIR spectroscopy, and clinical techniques allowing *in vivo* analysis, such as MRI and PET, are invaluable in obtaining further understanding on the molecular mechanisms involved in the pathogenesis of AD and to confirm the diagnosis of AD through the identification of unique biomarkers present in the metabolome, lipidome and/or proteome. Additionally, the techniques described in this review have the potential to follow disease progression in AD patients from early to severe stages and assess the effect of novel therapeutic interventions which may retard, stop or reverse progressive neurodegeneration, the ultimate goal being a cure for this debilitating neurodegenerative disorder.

## Author contributions

Nady Braidy, Christopher Marjo, Anne Poljak, Tharusha Jayasena, Nibaldo C. Inestrosa, and Perminder Sachdev wrote the draft, reviewed and interpreted the bioimages. Helen Rutlidge and Anne Rich processed the images. Nibaldo C. Inestrosa and Perminder Sachdev provided the conceptual foundation of the review, writing of drafts and interpretation of data.

### Conflict of interest statement

The authors declare that the research was conducted in the absence of any commercial or financial relationships that could be construed as a potential conflict of interest.
